# Prophylaxis of Contrast-Induced Nephrotoxicity

**DOI:** 10.1155/2014/308316

**Published:** 2014-04-10

**Authors:** Ulla Ludwig, Frieder Keller

**Affiliations:** Division of Nephrology, Internal Medicine I, University of Ulm, Albert-Einstein Allee 23, 89081 Ulm, Germany

## Abstract

Contrast-induced nephrotoxicity (CIN) is a form of acute kidney injury that follows intravascular contrast media exposure. CIN may be preventable because its risk factors are well established and the timing of renal insult is commonly known in advance. However, contrast-induced nephrotoxicity is still the third leading cause of iatrogenic renal failure. This important complication accounts up to 10% of acute renal failure cases in hospitalized patients and it is associated with increased short- and long-term morbidity and mortality. Prolonged hospitalization follows and overall increases healthcare resource utilization. This paper will discuss the various prophylactic procedures tested in clinical trials.

## 1. Introduction

The general indication for the use of radiographic contrast agents is to enhance images in diagnostic and therapeutic interventions. Increasing use of contrast media (CM) during radiological procedures has resulted in an increasing incidence of contrast-induced nephrotoxicity (CIN). In the year 2003, about 8 million liters of contrast media was used in 80 million contrast media examinations [1]. This makes it one of the highest volumes of medical drugs used. Development of contrast-induced nephrotoxicity (CIN) is a common complication of radiocontrast media exposure in patients who possess underlying risk factors.

## 2. Definition of CIN

The definition of CIN varies widely and refers to the development of acute renal impairment following the intravascular administration of radiocontrast dye in the absence of other identifiable causes of renal failure. Typically it occurs within 24–48 hours after administration of contrast media and peaks by day 5 after exposure [1, 2]. The most commonly used definition is an increase of more than ≥25% in serum creatinine level (SCr) or an absolute increase of 0.5 mg/dL (44.2 *μ*mol/L) from baseline value [1–5]. CIN corresponds to one stage increase in the 3 stages according to the KDIGO acute kidney injury network criteria [6].

## 3. Risk Factors for Contrast-Induced Nephrotoxicity

Risk factors for the development of CIN have been examined in several studies and can be divided into patient-related and non-patient-related factors. The patient-related risk factors include preexisting renal dysfunction, diabetes mellitus, multiple myeloma, advanced age, congestive heart failure, hemodynamic instability, hypertension, hypotension, emergency procedure, anaemia, left ventricular ejection fraction <40%, nephrotic syndrome, and myocardial infarction [3, 7]. The non-patient-related risk factors are volume, osmolality, ionicity and viscosity of the contrast media, intra-arterial versus intravenous injection, concomitant use of nephrotoxic drugs, and volume depletion [7, 8]. The most important risk factor for CIN is chronic kidney disease (CKD). Generally, the estimated glomerular filtration rate (eGFR) <60 mL/min/1.73 m^2^ is considered a cut-off value for increased risk for CIN (2%) [8]. The lower the eGFR value is, the greater the risk of CIN is. It is fivefold higher (10.2%), if serum creatinine is in the range 1.4–1.9 mg/dL [3]. The majority of the studies on CIN were performed on patients undergoing cardiac procedure following intra-arterial CM administration. Thus, one conclusion was therefore that intra-arterial CM administration leads to a higher risk of CIN as compared to an intravenous study population [9, 10]. The only one head-to-head study available to date comparing intravenous route with intra-arterial route found is the study of Karlsberg et al. This study showed that intravenous route might be as nephrotoxic as intra-arterial route; even the dose of applied CM was higher. So most of the intra-arterial injections are mainly intravenous for the kidney [11]. Volume of contrast media seems to be a major procedure-related risk factor of CIN. Therefore, the use of volume-to-creatinine clearance ratio (v/CrCl) can be used as an index for prediction of an abnormal increase in postinterventional creatinine. A ratio of the CM volume to the creatinine clearance below 2.62 has been suggested as a safe limit [12, 13]. But a “safe” dose does not exist and even very limited doses of CM may cause CIN in high-risk patients. The likelihood of CIN rises sharply as the number of risk factors increases. Cystatin C appears to be a good biomarker in the prediction of acute kidney injury, but so far it is almost not used to detect CIN [14]. Other new biomarkers such as neutrophil gelatinase-associated lipocalin (NGAL) are not helpful to better diagnose CIN [15]. Studies testing KIM-1 are inconsistent because of the small number of studies and heterogeneity between them [16].

## 4. Prevention for CIN

### 4.1. Hydration

Hydration only is the intervention best supported by evidence with a preventive effect on CIN, though no randomized controlled trials directly compared hydration versus no hydration. Intravenous hydration seems to be more effective than unrestricted oral hydration [17]. Standardized prospective studies to determine the optimal hydration strategy are needed. Several potential mechanisms can contribute to the beneficial effect of volume expansion, including dilution of contrast media within the tubule lumen, increased diuresis, reduced activation of renin-angiotensin system due to increased delivery of sodium to the distal nephron, and minimizing of the renal production of nitric oxide [18]. Solomon et al. were the first who showed the positive effect of adequate hydration [19]. Contrasting the Solomon study, others and we found that furosemide was beneficial. Furosemide was given after CM and not before it in our study; in the other studies the urine output rate should be >300 mL/h [20, 21]. The high urine output and positive fluid balance in combination with furosemide to keep the high-risk patients euvolemic is controlled by the RenalGuard system. These studies are promising but investigator driven [23, 24]. In addition to timing and route of hydration, other factors, such as fluid composition, may also play a role. In a randomized trial that included 1620 patients, Mueller et al. showed that intravenously administered 0.9% saline solution was superior to 0.45% saline solution [22]. Furthermore, two small studies suggest that sustained fluid administration intravenously within 12 h before and 12 h after administration of contrast media is superior to bolus administration at the time of contrast administration [25, 26]. CIN Consensus Working Panel recommendations published in 2006 suggested adequate intravenous volume expansion with isotonic crystalloid (1.0–1.5 mL/kg/hr) for 3 to 12 hr before the procedure and continued for 6 to 24 hr to prevent CIN in patients at risk [27].

### 4.2. Vasodilators

Renal vasodilatators, including calcium-channel antagonists, are promising agents in the prevention of CIN. So far their administration has failed to show conclusive evidence of a beneficial effect [28, 29]. Given its dilatory effect on the renal vasculature and the ability to increase renal blood flow and GFR, dopamine was supposed to be useful in the prevention of CIN. This hypothesis was evaluated in several studies and none showed a benefit in terms of dopamine administration [30–32]. Fenoldopam, a selective dopamine-1 receptor agonist with vasodilatory properties, was unable to lower the risk of CIN in a small population [33–35]. Critical experts argue that the doses of dopamine and fenoldopam used in these trials may have been insufficient to produce renal vasodilatation [36]. The adverse effects of these drugs were arrhythmia with dopamine and hypotension associated with intravenous fenoldopam administration. Small underpowered trials using vasodilating agents such as natriuretic peptide [37], an endothelin antagonist [38], prostaglandin E_1_ [39], angiotensin converting enzyme inhibitors [40], and L-arginine [41] have shown no benefit and in some cases even a potential harm [41].

### 4.3. Sodium Bicarbonate

Sodium bicarbonate may be an effective therapy for the prevention of contrast-induced nephrotoxicity [42]. The proposed mechanisms are that alkalinizing the tubular urine with sodium bicarbonate infusion may attenuate free radical formation and oxidant injury. Merten et al. presented the first study to prevent CIN by the administration of bicarbonate solution in a concentration of 154 mMol/L. In this study, the administration of bicarbonate was associated with a decreased incidence of CIN [43]. Subsequent studies have failed to show any additional benefit of the intravenous administration of sodium bicarbonate over isotonic sodium chloride alone in CIN prevention; also these studies had a dose reduction of NaHCO_3_ [44, 45]. In a systematic overview of 14 randomized trials, 2290 patients were included comparing sodium bicarbonate with sodium chloride for the prevention of CIN. Of those trials three were categorized as large (*n* = 1145) and 12 as small (*n* = 1145). Among the large trials, the CIN incidence for sodium bicarbonate and sodium chloride was 10.7 and 12.5%, respectively; the relative risk (RR) with 95% confidence interval (CI) was 0.85 (0.63 to 1.16) without evidence of heterogeneity (*P* = 0.09, *I*(2) = 0%). The pooled RR (95% CI) among the 12 small trials was 0.50 (0.27 to 0.93) with significant between-trial heterogeneity (*P* = 0.01; *I*(2) = 56%). The small trials were more likely to show a benefit for hydration with sodium bicarbonate, but these studies were generally of lower methodological quality. Among the larger, randomized trials, there was no statistically significant difference between hydration with sodium bicarbonate and sodium chloride. These data suggest that the true clinical benefit of hydration with sodium bicarbonate, if any, is likely to be small for the average patient [46–50].

### 4.4. Antioxidant: N-Acetylcysteine

The use of N-acetylcysteine, an agent with antioxidant properties, in the prevention of CIN is based on the assumption that CIN is caused by reactive oxygen species (ROS). ROS presumably are formed as a result of direct toxic effect of contrast media on tubular epithelial cells. Tepel et al. conducted the first study [51], showing that serum creatinine levels rose by more than 0.5 mg/dL in only 2% of patients who received N-acetylcysteine (600 mg bid orally) as compared to 21% of patients in the control group (*P* < 0.001). In the control group, 9 patients needed dialysis but only 1 in the N-acetylcysteine group. Many other studies on N-acetylcysteine followed; one of the latest is the large study of Berwanger and the ACT Investigators published in 2011. This study showed no benefit using N-acetycysteine p.o. in the incidence of CIN reduction as well as other clinically relevant outcomes [52]. Several meta-analyses showed no significant benefits of N-acetylcysteine (600 mg bid orally) compared to controls [53, 54]. Thus, the meta-analyses of N-acetylcysteine trials have led to disparate conclusions. The latest report included 22 trials with 2746 patients. There was a significant heterogeneity among those trials (*I* (2) = 37%; *P* = 0.04), but meta-regression analysis failed to identify significant sources of heterogeneity. Two clusters were studied: cluster 1 (*n* = 18; 2445 patients) showed no benefit where the relative risk (RR) was 0.87 and the 95% confidence interval (CI) 0.68–1.12 (*P* = 0.28). The studies in cluster 2 (*n* = 4; 301 patients) indicated that N-acetylcysteine was highly beneficial (RR = 0.15; 95% CI 0.07–0.33, *P* < 0.0001). However, cluster 2 studies were relatively early, small, and of lower quality compared with cluster 1 studies (*P* = 0.01 for the three factors combined). Need for dialysis across all studies (5 in control group and 8 in the treatment group, *P* = 0.42) did not suggest that N-acetylcysteine is beneficial [55, 56]. The dose of N-acetylcysteine that has been investigated might be too low to achieve meaningful ROS reduction. Briguori et al. compared therefore standard dose (600 mg bid orally) versus high doses (1200 mg bid orally) on the day of procedure [57]; the rate of CIN was lower in patients receiving high-dose N-acetylcysteine (4% versus 11%; *P* = 0.03). The benefit of high-dose N-acetylcysteine versus intravenous hydration was even more pronounced in the study by Baker et al., where N-acetylcysteine was given intravenously immediately before contrast agent [58]. CIN occurred in 2 patients in the N-acetylcysteine group (5%) and in 8 patients in the hydration group (21%, *P* = 0.045). Therefore, this high intravenous dose protocol can be used for all emergency patients or outpatients at the same day. Further investigations for such protocols are needed (Table 1).

### 4.5. Antioxidant: Mesna

**Figure 1:**
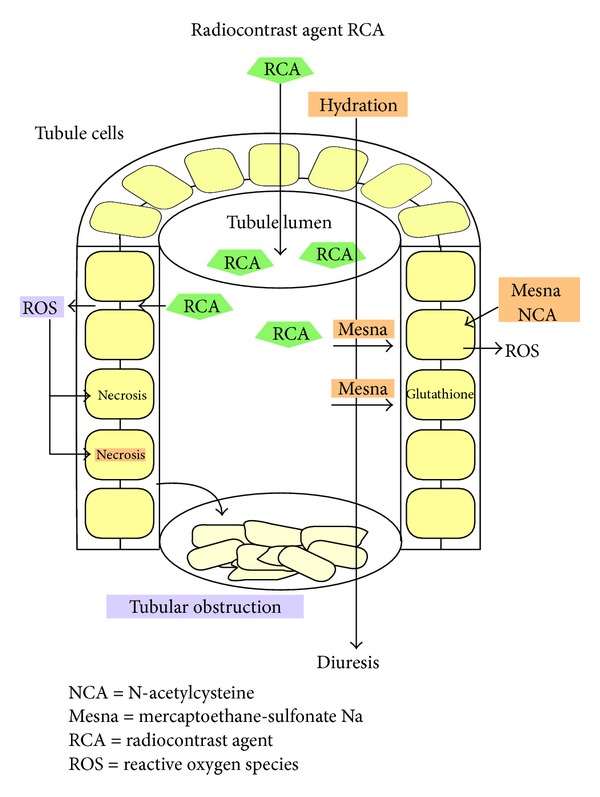
Presumed renoprotective effects of Mesna in tubule lumen. Radiocontrast agents (RCA) are filtered into the primary urine. In the tubule lumen, RCAs are concentrated 100-fold by water reabsorption. High RCA concentration stimulates the production of reactive oxygen (ROS) in the tubule cells leading to epithelial cell necrosis and tubule obstruction. By hydration, RCAs are less concentrated and tubule obstruction can be washed out. While N-acetylcysteine acts mainly from the basal side, Mesna can prevent ROS production in the tubule cells from both apical and basal sides. Finally Mesna may prevent contrast-agent-induced nephrotoxicity by glutathione regeneration. Graphic: G.Hintze.

Mesna (mercaptoethane-sulfonate Na), an agent with antioxidant properties, can reduce free radicals and restore reduced glutathione (GSH) levels after ischemic renal failure [61]. An advantage of Mesna is its 60% elimination by glomerular filtration (Figure 1), whereas N-acetylcysteine is less than 10% excreted in the urine [59]. There is so far only one single randomized controlled trial by our group, which investigated the use of Mesna in prevention of contrast-induced nephrotoxicity. In our study we compared the efficacy of intravenous administration of 1600 mg Mesna versus placebo in addition to intravenous hydration with 0.9% saline. The results were a CIN in 7 patients in the placebo group and none in the Mesna group. The immediate preinvestigational infusion makes Mesna easy to use in outpatients as well as for emergency procedures. Clearly, the investigation by a multicenter trial is needed to confirm the benefit of Mesna.

### 4.6. Antioxidant: Ascorbic Acid

Ascorbic acid can reduce free radical production. Oral ascorbic acid (3 g before and 2 g twice after the procedure) was evaluated in a randomized controlled trial that included 231 patients [62]. The incidence in contrast-induced nephrotoxicity was 9% in the ascorbic acid group and 20% in the placebo group (*P* = 0.02). In a recently published meta-analysis Sadat et al. showed that in 1536 patients, who completed the trial, ascorbic acid produced a 33% lower risk of developing a CIN [63]. So ascorbic acid might be a form of prophylactic regime in contrast media induced renal failure; nevertheless it has not been recommended by the CM safety committee.

### 4.7. Theophylline

There are only small studies of theophylline as a potential prophylactic agent for CIN with conflicting results. Nine trials (*n* = 585 patients) compared theophylline with no active treatment. Meta-analysis identified considerable heterogeneity among these studies [60]. There was variability in the inclusion criteria, the method, and schedule of theophylline administration and hydration protocols as well as in the type of contrast media. Only few trials compared the incidence of adverse events. To date there is no supporting evidence for the use of theophylline for the prevention of CIN.

### 4.8. Atrial Natriuretic Peptide

Atrial natriuretic peptide failed to prevent CIN in a randomized, placebo-controlled study of Kurnik et al. [37].

### 4.9. Statins

Statins have been shown to have pleiotropic, antioxidant, and anti-inflammatory effects. In a retrospective register study of 29409 patients who underwent percutaneous coronary angiography, statin therapy before the procedure was linked with a lower incidence of CIN compared to patients not taking a statin at that time [64]. These results are in line with a prospective, observational study that included 434 participants. Patients who were taking statins before undergoing coronary angiography had a lower rate of CIN [65]. However, later ongoing trials with simvastatin in the PROMISS study [66] and in the diabetes subgroup failed to demonstrate benefits of treatment as well as with atorvastatin [67]. Another trial has been started with high-loading dose of atorvastatin (80 mg); in this small study the results show a benefit for patients receiving the high-loading dose compared to placebo (CIN: 5% versus 13.2%, *P* = 0.046) [68]. In the multivariable analysis, atorvastatin pretreatment was independently associated with a decreased risk of CIN (odds ratios 0.34, 95% confidence interval 0.12 to 0.97, *P* = 0.043) and shortening of hospital days [68]. Quintavalle et al. demonstrated in a single centre prospective study with 410 patients that a single high-loading dose of atorvastatin (80 mg within 24 hours before contrast media exposure) significantly reduced the risk of contrast-induced nephropathy (4.5% versus 17.8%). This effect was only obvious in patients with moderate risk and a glomerular filtration rate between 31 and 60 mL/min. The definition of contrast-induced renal failure is new, since cystatin C as marker was used [69]. The study was not powered to detect an effect according to the more traditional and less sensitive definition of contrast-induced acute kidney injury [70]. Another interesting approach was made with the substance rosuvastatin. Leoncini et al. could demonstrate that a high-dose rosuvastatin (40 mg given on admission to statin-naïve patients with ACS, followed by 20 mg/day) compared to no statin treatment reduced the risk of CIN significantly (6.7% versus 15.1%; adjusted odds ratio: 0.38; 95% confidence interval (CI): 0.20 to 0.71; *P* = 0.003). In this interesting study the benefit of rosuvastatin was consistent, even applying different definitions of contrast-induced nephropathy. It showed even after 6-month follow-up further benefit with lower rate of death or nonfatal myocardial infarction [71].So since the substances are heterogeneous as well as the dose, further investigations are needed.

### 4.10. Hemodialysis and Hemofiltration

Several studies examined the effect of hemodialysis, immediately after exposure to contrast media to prevent the further deterioration of renal function in patients with preexisting advanced renal disease. Theoretically, hemodialysis is an effective method in removing contrast media from the patient's body. One study of removing contrast media via hemodialysis was even performed during coronary intervention in patients with advanced renal insufficiency, but no significant effect on renal function was observed compared with patients who did not undergo hemodialysis [72]. Paradoxically, in a study reported by Vogt et al. hemodialysis performed after CM administration was associated with a significantly greater mean peak in serum creatinine (*P* < 0.05) compared with patients who did not undergo hemodialysis [73, 74]. Finally, patients with end-stage renal failure who underwent a 6-hour hemodialysis postprocedure were without a benefit [75]. On the basis of these data, hemodialysis cannot be recommended.

Two studies [76, 77] investigated the effect of continuous venovenous hemofiltration for prevention of CIN in patients with chronic renal insufficiency as compared with intravenous hydration. A >25% increase of creatinine and the in-hospital mortality were significantly lower in the hemofiltration group. However, since creatinine level is naturally influenced by hemofiltration, assessment of benefit in prevention on CIN based on this endpoint is certainly debatable. The benefits of hemofiltration observed in this study have been suggested to be due to the concomitant administration of heparin, which has anti-inflammatory effects and might reduce ROS generation. A different mechanism that might play a role was high-volume controlled hydration before contrast media exposure [77]. This method deserves further investigations; nevertheless, it is expensive and invasive.

## 5. Contrast Media Use

Contrast media are classified according to osmolality, which reflects the total particle concentration of the solution. High-osmolar contrast media (HOCM) have about 1500 to 1800 mOsmol/kg osmolality. Low-osmolar contrast media (LOCM) have 600 to 800 mOsm/kg and isoosmolar contrast media (IOCM) 290 mOsm/kg. In a meta-analysis of comparative trials [78], an increase in serum creatinine of more than 0.5 mg/dL after administration of contrast media was less frequent with low-osmolar than with high-osmolar contrast media (odds ratio, 0.5; 95% confidence interval, 0.36 to 0.68). Thus in western countries HOCM have been completely replaced by LOCM due to the lower incidence of side effects from LOCM with no difference in image quality. In a recently published systematic overview of 36 randomized, controlled trials (*n* = 7166 patients) nephrotoxicity of isoosmolar contrast media iodixanol (*n* = 3672) was compared to diverse low-osmolar contrast media (*n* = 3494). In this analysis [79], iodixanol showed no statistically significant reduction in the incidence of contrast-induced nephrotoxicity below that observed with heterogeneous comparator agents. Since molecular weight of isoosmolar agents is higher (1550 daltons versus 750–850 daltons in low-osmolar agents), they have a higher viscosity and likely therefore no significant benefit could be shown in any high-risk subgroups; there was only a significant benefit of iodixanol when compared with iohexol [79]. So the CM safety committee (GMSC) guidelines recommend the use of LOCM and IOCM in patients with risk factors for CIN [80].

## 6. Summary and Recommendations

Contrast-induced nephrotoxicity is a serious adverse event for which preventive care is needed since treatment options are of limited value. Physicians using contrast media should incorporate preventive strategies into their clinical practices. The consulting nephrologists can provide guidance to radiologists and cardiologists regarding the identification of patients at risk and suggest the best practical strategy to reduce the incidence of CIN.

These practical measures include the assessment of patients at risk. Patients with normal kidney function and without risk factor for contrast-induced nephrotoxicity do not require prophylactic intervention before contrast media use. Patients with underlying renal dysfunction and an estimated glomerular filtration rate (eGFR) <60 mL/min/1.73 m^2^ have to be identified particularly in combination with other risks which have to be identified. Potentially nephrotoxic drugs (NSAIDs) as well as metformin should be withdrawn before contrast administration. The use of the lowest contrast volume as possible is recommended and high-osmolar contrast media should be avoided. The best way to prevent CIN is to provide adequate periprocedural hydration. The role of various drugs in prevention of CIN (such as Mesna) is still controversial and warrants future studies.

## Figures and Tables

**Table 1:** Agents and measures proposed for prevention of contrast-agent-induced nephrotoxicity.

Drug	Trial	Patients	Prophylactic benefit	Reference
Mesna	RCT	*N* = 100	Yes	[59]

NAC	RCT Meta-analysis	*N* = 83 *N* = 2746	Yes Equivocal	[51] [55, 56]

Hydration	RCT RCT	*N* = 78 *N* = 1620 *N* = 53	Yes Yes Yes	[19] [22] [17]

Sodium bicarbonate	RCT RCT Meta-analysis	*N* = 119 *N* = 353 *N* = 2290	Yes No Equivocal	[43] [44] [46]

Theophyllin	Meta-analysis	*N* = 585	No	[60]
